# Identification and expression analysis of ATP-binding cassette (ABC) transporters revealed its role in regulating stress response in pear (*Pyrus bretchneideri*)

**DOI:** 10.1186/s12864-024-10063-1

**Published:** 2024-02-12

**Authors:** Xiaobing Kou, Zhen Zhao, Xinqi Xu, Chang Li, Juyou Wu, Shaoling Zhang

**Affiliations:** 1 School of Life Sciences, Nantong University, Nantong, 226019 Jiangsu People’s Republic of China; 2 Centre of Pear Engineering Technology Research, State Key Laboratory of Crop Genetics and Germplasm Enhancement, College of Horticulture, Nanjing Agricultural University, Nanjing, 210095 China

**Keywords:** *Pyrus bretchneideri*, ABC transporter, Expression file, Salt stress, Drought stress

## Abstract

**Background:**

ATP-binding cassette (ABC) transporter proteins constitute a plant gene superfamily crucial for growth, development, and responses to environmental stresses. Despite their identification in various plants like maize, rice, and Arabidopsis, little is known about the information on ABC transporters in pear. To investigate the functions of ABC transporters in pear development and abiotic stress response, we conducted an extensive analysis of ABC gene family in the pear genome.

**Results:**

In this study, 177 ABC transporter genes were successfully identified in the pear genome, classified into seven subfamilies: 8 ABCAs, 40 ABCBs, 24 ABCCs, 8 ABCDs, 9 ABCEs, 8 ABCFs, and 80 ABCGs. Ten motifs were common among all ABC transporter proteins, while distinct motif structures were observed for each subfamily. Distribution analysis revealed 85 *PbrABC* transporter genes across 17 chromosomes, driven primarily by WGD and dispersed duplication. *Cis*-regulatory element analysis of *PbrABC* promoters indicated associations with phytohormones and stress responses. Tissue-specific expression profiles demonstrated varied expression levels across tissues, suggesting diverse functions in development. Furthermore, several *PbrABC* genes responded to abiotic stresses, with 82 genes sensitive to salt stress, including 40 upregulated and 23 downregulated genes. Additionally, 91 genes were responsive to drought stress, with 22 upregulated and 36 downregulated genes. These findings highlight the pivotal role of *PbrABC* genes in abiotic stress responses.

**Conclusion:**

This study provides evolutionary insights into *PbrABC* transporter genes, establishing a foundation for future research on their functions in pear. The identified motifs, distribution patterns, and stress-responsive expressions contribute to understanding the regulatory mechanisms of ABC transporters in pear. The observed tissue-specific expression profiles suggest diverse roles in developmental processes. Notably, the significant responses to salt and drought stress emphasize the importance of *PbrABC* genes in mediating adaptive responses. Overall, our study advances the understanding of *PbrABC* transporter genes in pear, opening avenues for further investigations in plant molecular biology and stress physiology.

**Supplementary Information:**

The online version contains supplementary material available at 10.1186/s12864-024-10063-1.

## Introduction

ATP-binding cassette (ABC) transporters constitute a versatile family of membrane proteins that play pivotal roles in the transport of various substrates across cellular membranes [1–3]. These transporters are characterized by a conserved structural motif comprising two transmembrane domains (TMDs) and two nucleotide-binding domains (NBDs), forming the core structure responsible for substrate translocation and ATP hydrolysis [4, 5]. ABC transporters are widely distributed across the domains of life, from prokaryotes to eukaryotes, and have been extensively studied for their significance in diverse biological processes [6–11].

In various species, extensive research has been devoted to ABC transporters due to their multifaceted roles [6, 9–14]. In mammals, they are renowned for their involvement in drug resistance, exemplified by the P-glycoprotein encoded by the *ABCB1* gene, which confers resistance to anticancer drugs [15–17]. In yeast, transporters like *Pdr5p* are critical for the efflux of xenobiotics, including drugs and toxins, thus contributing to cellular detoxification [18, 19]. In plants, ABC transporters have garnered substantial attention due to their vital functions in growth, development, and stress responses [12–14, 20]. For instance, the *ABCB19* gene in *Arabidopsis thaliana* encodes a transporter that regulates the polar transport of the phytohormone auxin, crucial for plant development and tropisms [21–24]. In addition, Arabidopsis ABCG27 plays an essential role in flower and leaf development by modulating abscisic acid content [25]. In wheat, the suppression of the ABCC13 transporter unveils its role in grain development, the accumulation of phytic acid, and the formation of lateral roots [26]. Furthermore, the ATP-binding cassette (ABC) transporter OsABCG3 is essential for pollen development in rice [27]. These examples illustrate the diversity of roles ABC transporters play in plant growth and development.

One of the most challenging aspects of plant growth is the ability to withstand environmental stressors such as salinity and drought. These stress factors can severely affect plant growth and yield. ABC transporters have emerged as key players in plant stress responses by facilitating the transport of toxic ions, heavy metals, and other stress-related molecules out of the cell, thereby alleviating the detrimental effects of stress [14, 20, 28]. In Arabidopsis, the *AtABCC1* gene encodes an ABC transporter involved in the detoxification of heavy metals, including cadmium and mercury [29]. Additionally, the *ABCG36* gene in rice plays a crucial role in exporting toxic heavy metals such as arsenic from roots to shoots, thereby reducing their accumulation in edible parts of the plant [20]. In *Artemisia annua*, AaABCG40 enhances artemisinin content and modulates drought tolerance [30]. Furthermore, ABC transporters have been implicated in temperature stress responses. For example, in *thellungiella salsugineum*, overexpression of *TsABCG11* exhibited higher photosynthetic rates and water-use efficiency under cold stress (4 °C) than control plants [31]. These findings underscore the pivotal role of ABC transporters in plant stress responses.

Pear (*Pyrus bretchneideri*) stands as an economically significant fruit crop with growing global importance. However, the genetic and molecular mechanisms underlying stress responses in pear remain insufficiently explored. Given the crucial roles of ABC transporters in stress mitigation and plant growth, comprehensively analyzing the ABC transporter family in pear holds immense potential for advancing our understanding of stress tolerance mechanisms and enhancing pear cultivation. This study embarks on a genome-wide identification and expression analysis of ABC transporters in pear to shed light on their biological functions, with a particular focus on their involvement in regulating stress responses. Through this investigation, we aim to provide a theoretical foundation for further research into the biological functions of ABC transporters in pear and their potential applications in stress-resistant pear breeding programs.

## Results

### Identification and characterization of ABC transporters in pear

For the genome-wide identification of ABC transporters in pear, we utilized the Hidden Markov Model (HMM) profile of the ABC transport (PF00005) as queries to search against the pear genome database. In addition, potential PbrABC members were extracted using the BLAST tool, which utilized the 131 Arabidopsis ABC proteins as queries against the pear genome. After removing redundant sequences, the SMART database was used to examine the presence of the ABC transport and ABC transmembrane domains for each identified candidate. As a result, a total of 177 *PbrABC* genes were identified in pear. The identified *PbrABC* genes encoded proteins ranging from a minimum of 160 (PbrABC) to a maximum of 847 (PbrABC) amino acids, with molecular weights varying between 20.29 and 277.34 kDa. Furthermore, the isoelectric points of these ABC proteins displayed considerable variation, spanning from 4.89 to 10.63. Chromosome mapping offers evidence that 177 *PbrABC* genes are nonrandomly distributed in the pear genome, with the fewest ABC genes located on chromosomes 4, only containing two genes, while the highest number of *PbrABC* genes was identified on chromosome 7, totaling 16 genes. The details for the *PbrABC*s, including the subgroup names, gene names, and gene IDs are summarized in Table S1.

### Phylogenetic and conserved motif analysis of pear ABC proteins

**Fig. 1:**
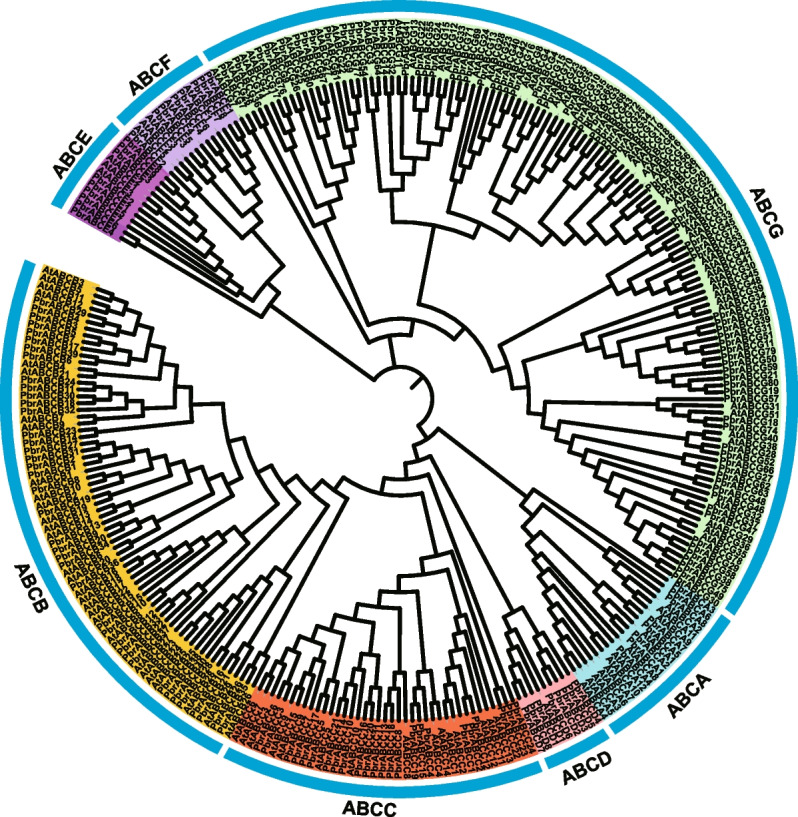
Phylogenetic relationships of ABC transporters in Arabidopsis and pear. Full-length ABC transporters sequences were aligned using the Clustal X software, and the neighbor-joining (NJ) phylogenetic tree was constructed using MEGA 7.0 with 1000 bootstrap replicates. Different subfamilies are highlighted in different colors

To investigate the evolutionary relationships of ABC transporters, we constructed a neighbor joining phylogenetic tree in MAGA 7.0 using the full-length protein sequences of 177 PbrABCs from pear and 144 AtABCs from Arabidopsis. According to the phylogenetic tree (Fig. 1), the ABC transporters from pear and arabidopsis were divided into seven major groups based on their similarities and relationship with 130 Arabidopsis members. Within the pear genome, the ABC transporter gene subfamilies exhibit distinct sizes: ABCA comprises 8 members, ABCB encompasses 40 members, ABCC consists of 24 members, ABCD is represented by 8 members, ABCE includes 9 members, ABCF has 8 members, and ABCG stands out as the most substantial subfamily with 80 members.

**Fig. 2:**
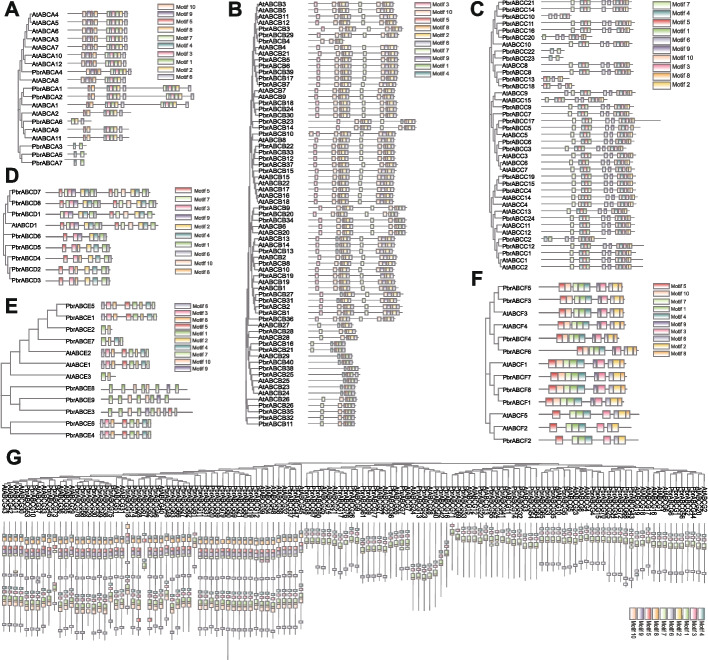
Distributions of conserved motifs in ABC transporters from Arabidopsis and pear. The motif of ABC transporters was analyzed by MEME tool. Ten motifs (1–10) were identified and are indicated by different colors

To unveil the diversification among pear ABC genes more effectively, we conducted an analysis of their conserved motifs. We employed the MEME online tool to predict the composition of conserved motifs within the PbrABCs (Fig. 2). The number of motifs ranged from 3 to 19. Notably, Motif 1, representing the ABC domain, was detected in all PbrABCs. The PbrABC proteins within each group exhibited significant similarity among orthologous members but displayed notable distinctions from members in the other groups, suggesting a divergent evolution from a shared ancestor or their origin from gene duplication events.

### Chromosomal distribution and collinearity analysis of *PbrABC* genes

**Fig. 3:**
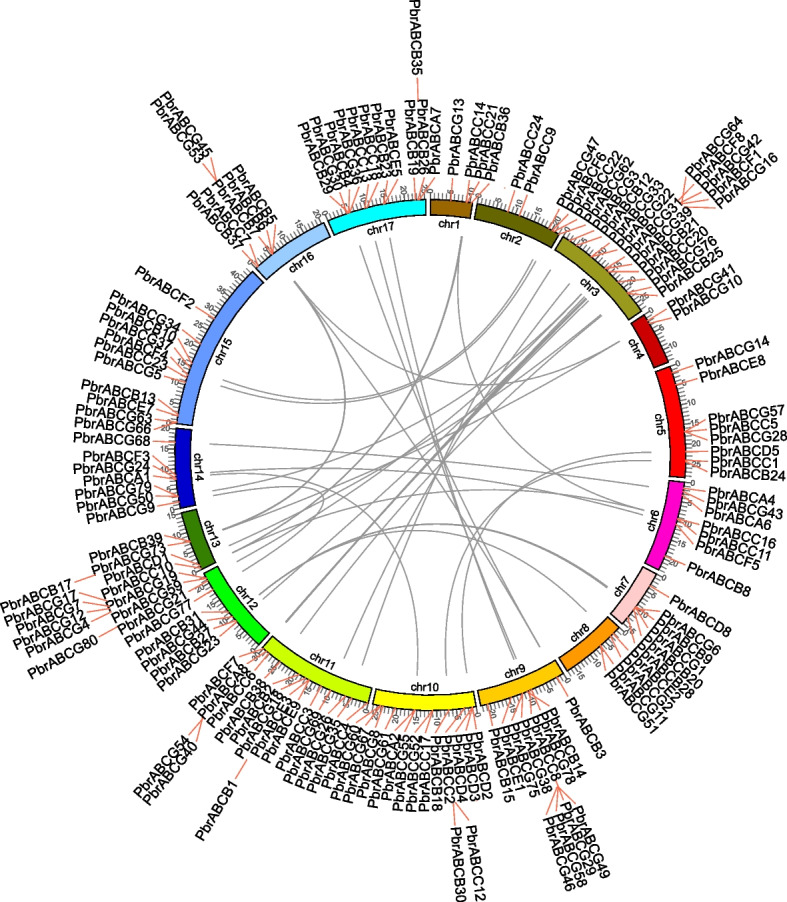
Chromosomal localization and syntenic relationships of ABC transporters in pear. PbrABC transporter genes are mapped on different chromosomes and syntenic gene pairs are linked by colored lines

Based on the genomic positions of 177 pear ABC genes on pear chromosomes, we assessed the chromosomal distribution of the *PbrABC* gene family (Fig. 3). The findings reveal that among these 177 *PbrABC* genes, 148 PbrABC genes are heterogeneously distributed across 17 chromosomes, displaying varying quantities on each chromosome. Notably, chromosome 11 emerges as the chromosome harboring the highest number of *PbrABC* genes, boasting a total of 16 members. Conversely, chromosome 4 hosts the fewest members, containing only 2 *PbrABC* genes. The remaining chromosomes exhibit distinct numbers of *PbrABC* gene members, such as chromosome 1 (4 members), chromosome 2 (6 members), chromosome 3 (15 members), chromosome 5 (8 members), chromosome 6 (7 members), chromosome 7 (6 members), chromosome 8 (4 members), chromosome 9 (12 members), chromosome 10 (15 members), chromosome 12 (13 members), chromosome 13 (5 members), chromosome 14 (7 members), chromosome 15 (7 members), chromosome 16 (7 members), and chromosome 17 (12 members). This comprehensive distribution analysis underscores the chromosomal variations in the abundance of PbrABC genes across the pear genome. Moreover, it’s intriguing to note that numerous *PbrABC* genes are arranged into clusters of different sizes, signifying occurrences of gene duplication during the evolutionary journey of *PbrABC* genes. The mechanisms potentially contributing to gene expansion during evolution encompass whole-genome duplication, tandem repeats, and dispersed duplication [32]. These mechanisms are pivotal in enabling functional diversification and gene separation. Scrutinizing the patterns of gene duplication is imperative for comprehending the evolution of gene families.

To reveal the expansion mechanisms of *PbrABC* genes within the pear genome, we conducted an analysis of gene duplication patterns. The results indicate that in the pear genome, 177 *PbrABC* genes partake in 84 (47%) WGD duplications and 64 (36%) dispersed duplications (Fig. S1), hinting at WGD and dispersed duplication as the predominant driving force behind the expansion of the ABC family in pear. Furthermore, our analysis highlights that chromosomes 3, 10, 11, 12 and 17 exhibit the highest repetition rates, which likely account for the increased abundance of *PbrABC* genes on these chromosomes. Identifying homologous genes is important for understanding the evolutionary history of gene families. To delve deeper into the origins and evolutionary relationships of *PbrABC* genes in pear, we conducted a collinearity analysis (Fig. 3). The outcomes unveil 48 pairs of collinear genes among *PbrABC* genes in the pear genome, with a heightened concentration of ABC homologous genes identified on pear chromosome 11. Consequently, this analysis offers valuable insights into the evolution and expansion of the pear ABC gene family, tailored to the linguistic standards of botanists and biologists.

### Analysis of *cis*-acting elements in the promoter region of pear ABC gene family

**Fig. 4:**
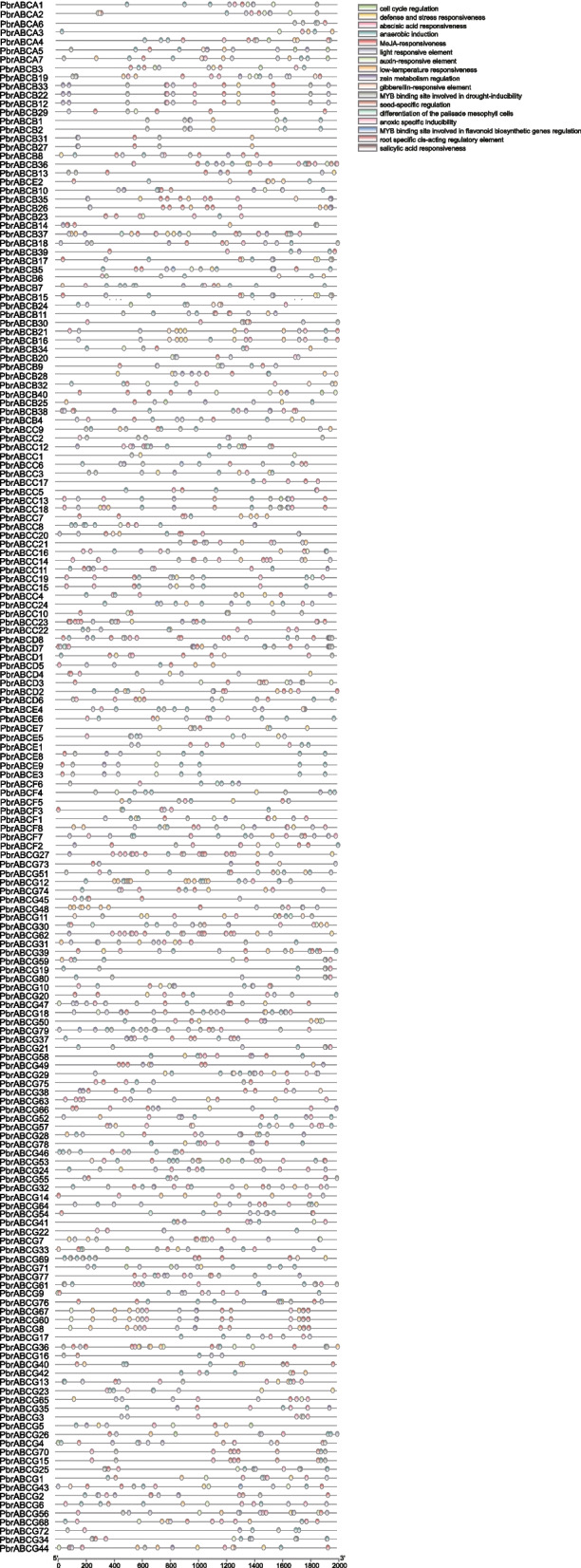
*Cis*-regulatory element analysis of PbrABC transporter genes. The *cis*-acting elements were predicted in the promoter sequences of the PbrABC transporter genes. Rectangular boxes of distinct colored boxes represent the different types of *cis*-acting elements

Cis-regulatory elements (CREs) are a family of non-coding DNA molecules that influence the transcription of neighboring genes, thereby controlling gene expression during different developmental stages [33, 34]. To gain a deeper understanding of the transcriptional regulation mechanisms of *PbrABC* genes, we characterized cis-regulatory elements within the 2000 bp upstream region of transcription start sites using the PlantCARE and PLACE databases (Fig. 4). Our results reveal that these identified cis-regulatory elements can be categorized into four primary functional groups: light response, hormone response, developmental regulation, and stress response. Notably, all 177 *PbrABC* genes contain at least one hormone-responsive element, including auxin and gibberellin-responsive elements. Furthermore, 150 cis-regulatory elements are associated with responses to abscisic acid, methyl jasmonate, ethylene, and salicylic acid, highlighting the crucial role of the PbrABC gene family in complex hormone regulatory networks. In addition, the promoter sequences of some *PbrABC* genes also harbor elements related to both biotic and abiotic stress responses, including defense elements (rich in AT and TC repeats), drought-responsive elements (MBS), cold-responsive elements (DRE and LTR), and anaerobic stress elements (ARE). This suggests potential functions of *PbrABC* genes in biological and abiotic stress responses. These findings enhance our understanding of the regulatory mechanisms of *PbrABC* genes, particularly their roles in plant growth and stress responses.

### Tissue-specific expression of *PbrABC* genes

**Fig. 5:**
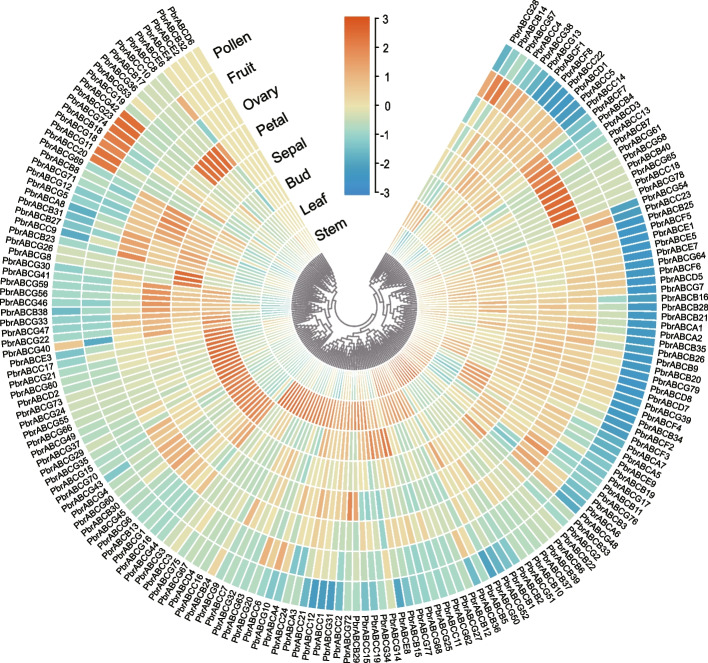
Expression file of PbrABC transporter genes in pear different tissues. Relative expression of PbrABC transporter genes in stem, leaf, bud, sepal, petal, overy, pollen and fruit were determined by RNA-Seq data from pear. Blue indicates low expression, and red indicates high expression. The heatmap was generated with TBtools

The gene expression patterns can provide direction and strategies for predicting gene biological functions. In order to explore the biological functions of *PbrABC* genes in pear, we downloaded transcriptome data from eight different tissues of pear from a public RNA-seq database and used it for a systematic analysis of expression patterns in the following tissues: stem, leaf, bud, petal, fruit, pollen, ovary, and sepal. The results of the analysis, as shown in Fig. 5, reveal that most PbrABC genes exhibit constitutive expression patterns. For instance, seven genes are significantly expressed in pear pollen (*PbrABCC20*, *PbrABCG11*, *PbrABCG18*, *PbrABCB18*, *PbrABCG74*, *PbrABCG23*, *PbrABCG42*), indicating potential crucial roles for these seven *PbrABC* genes in the growth and development of pear pollen tubes. In leaf tissue, nine *PbrABC* genes (*PbrABCA4*, *PbrABCC3*, *PbrABCC6*, *PbrABCC7*, *PbrABCD4*, *PbrABCG9*, *PbrABCG20*, *PbrABCG32*, *PbrABCG75*) exhibit a pronounced tissue-specific expression pattern, suggesting their involvement in leaf development. Forty-nine *PbrABC* genes show high expression in petals, implying their potential role in the development of floral organs.

### Expression analysis of PbrABCs in response to drought and salt stress

**Fig. 6:**
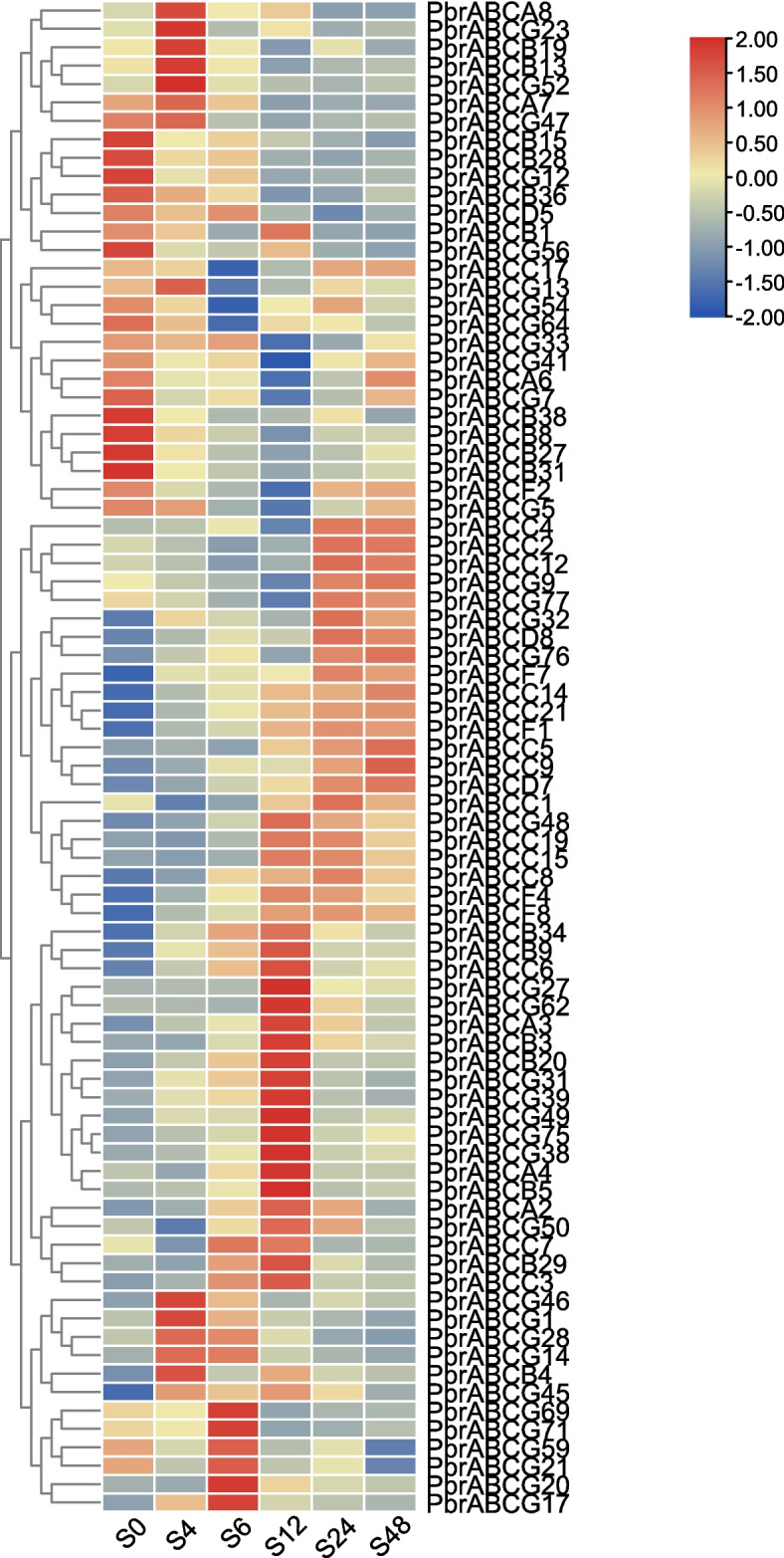
Expression levels of PbrABC transporter genes under salt stress. RNA-seq data were used to measure the expression level of PbrABC transporter genes under salt treatment. Blue indicates a low expression level and red indicates a high expression level. The heatmap was generated using TBtools

**Fig. 7:**
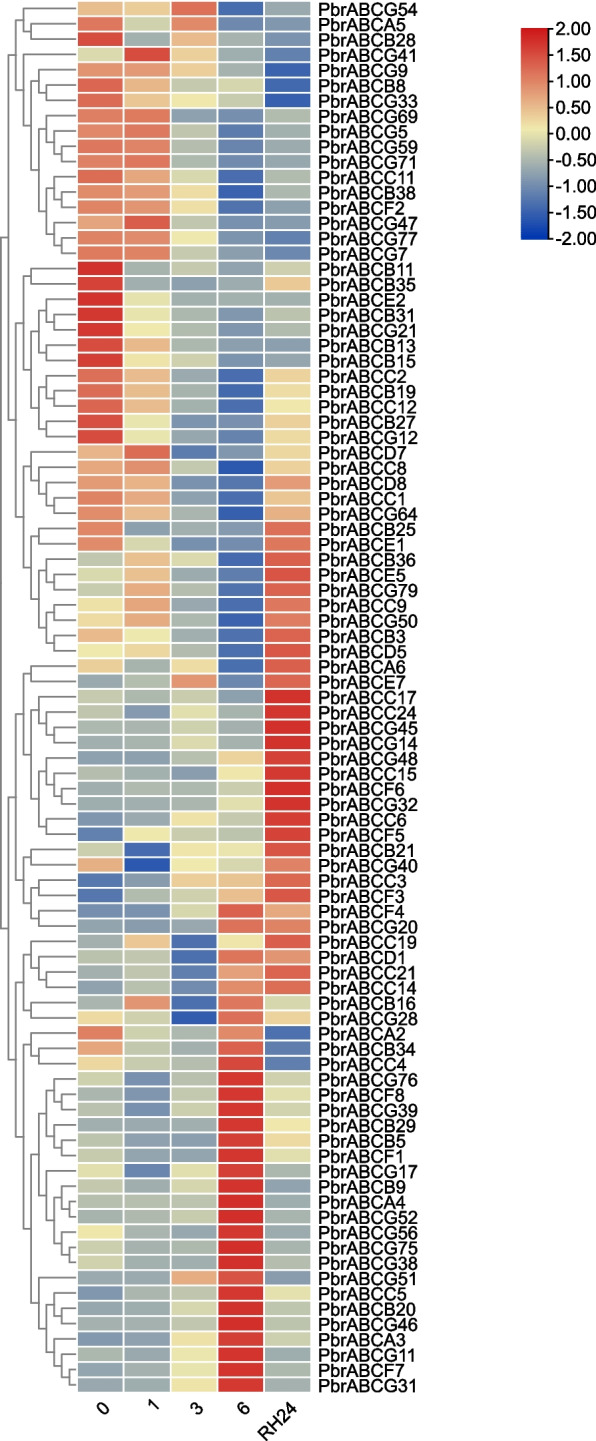
Expression levels of PbrABC transporter genes under drought stress. RNA-seq data were used to measure the expression level of PbrABC transporter genes under drought treatment. Blue indicates a low expression level and red indicates a high expression level. The heatmap was generated using TBtools

**Fig. 8:**
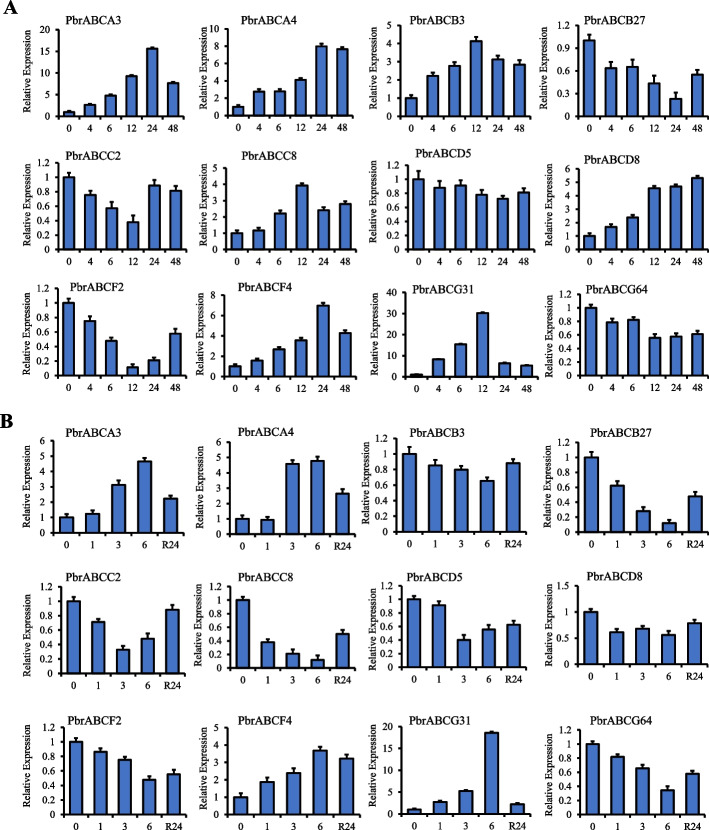
qRT–PCR analysis of 12 PbrABC transporter genes under salt and drought stress. **A**. Expression pattern of 12 PbrABC transporter genes under salt treatment for 0 h, 4 h, 6 h, 12 h, 24 h and 48 h, respectively. **B**. Expression pattern of 12 PbrABC transporter genes under drought treatment (10% PEG6000) for 0 h, 1 h, 3 h, 6 h and rehydration for 24 h, respectively. All experiments were performed independently at least three times

The ABC transporters have been reported to be widely involved in response to abiotic stress [20, 29, 31]. To investigate the expression patterns of the pear ABC transporters in response to abiotic stress, we conducted an analysis of expression changes in pear leaf tissues under drought and salt stress, based on the RNA-Seq data. As shown in Fig. 6, for salt stress, 82 of 177 *PbrABC* genes were sensitive to salt stress, the majority of which exhibited different expression profiles at different stages of salt treatment. For example, 21 *PbrABC* genes tended to be observably up-regulated at 12 h and decreased at 24 h. After salt treatment, *PbrABCC9*, *PbrABCC14*, *PbrABCC21*, *PbrABCD7* and *PbrABCF1* are continuously up-regulated. Interestingly, in the context of salt treatment, several *PbrABC* members exhibit expression trends resembling peaks or ‘V’-shaped expression patterns. For instance, 19 *PbrABC* genes exhibit an initial upward trend in expression, followed by either stabilization or a decline, whereas 12 *PbrABC* genes initially experience a continuous decrease in expression, subsequently stabilizing or increasing, this may be attributed to a complex adaptive strategy that plants employ in response to salt stress. As shown in Fig. 7, 91 *PbrABC* genes were responsive to the drought stress. Under drought treatment, the expression of 8 *PbrABC* genes increased first, then decreased after water recovery, while 17 *PbrABC* genes decreased first, then increased after water recovery. To validate the results obtained from the RNA-seq data, 12 *PbrABC* genes were selected for further validation by qRT–PCR. As shown in Fig. 8A, B, the expression trends of selected *PbrABC* genes from the qRT–PCR results were consistent with the RNA-seq data. The expression profiles of six selected PbrABC transporter genes (*PbrABCA3*, *PbrABCA4*, *PbrABCB3*, *PbrABCC8*, *PbrABCF4*, and *PbrABCG31*) were upregulated at 12 h or 24 h (0 h-12 h or 0 h-24 h) and then gradually decreased, with the expression of three *PbrABC*s (*PbrABCB27*, *PbrABCC2*, and *PbrABCF2*) were downregulated and then increased under salt treatment. For drought treatment, the expression levels of four genes (*PbrABCA3*, *PbrABCA4*, *PbrABCF4*, and *PbrABCG31*) were upregulated at 3 h or 6 h, and then decreased after rehydration, with eight genes (*PbrABCB3*, *PbrABCB27*, *PbrABCC2*, *PbrABCC8*, *PbrABCD5*, *PbrABCD8*, *PbrABCF2*, and *PbrABCG64*) were downregulated at 3 h or 6 h, and then increased after rehydration. The qRT–PCR results generally confirmed the RNA-seq results. Taken together, the diverse expression patterns observed suggest potential functional distinctions among *PbrABC* genes in response to abiotic stress.

## Discussion

ABC transporters are a multifunctional family of proteins found across various organisms, playing critical roles in the transport of a wide range of substrates [2–4]. In plants, these transporters have been implicated in the translocation of phytohormones, lipids, secondary metabolites, and ions, thus influencing plant growth, development, and stress responses [12, 13, 20–31]. The number of ABC transporters have been extensively studied in many species, such as *Arabidopsis thaliana* [25, 35, 36], *Fragaria vesca* [37], *Linum usitatissimum* [38], *Zea mays* [39] and *Oryza sativa* [20, 40]. However, the knowledge of the ABC gene family is very limited in pear. In the present study, we conducted an extensive analysis of ABC gene family in pear genome, a total of 177 ABC gene family members of *Pyrus* were identified genome wide, localized on 17 chromosomes (Fig. 3). The number of ABC transporter genes in *Pyrus* was more than that observed in the other model species such as *Arabidopsis thaliana* [41], *Oryza sativa* [42], *Solanum lycopersicum* [43] and *Zea mays* [39], this may be due to recent whole-genome duplication (WGD) events occurred in pear [44]. Phylogenetic results showed that these PbrABC transporters were divided into seven subfamilies (Fig. 1), of which the PbrABCG subfamily contains the most members in Pyrus. Furthermore, Members of the same subfamily have similar sequence lengths and motif distributions, the presence of conserved motifs within PbrABC proteins suggests the conservation of structural domains critical for their functions (Fig. 2).

Gene duplication is a prevalent mechanism occurred in a wide range of organisms, which furnish a reservoir of novel genes enables organisms to accommodate themselves to new environments. Whole-genome duplication (WGD), tandem duplication (TD), proximal duplication (PD), transposed duplication (TRD), and dispersed duplication (DSD) are the primary forms of gene duplication [32]. In this study, duplication analysis showed that most PbrABC transporter members in pear were duplicated from WGD or dispersed duplication events (Fig. S1), suggesting the WGD or dispersed duplications contributed to the expansion of PbrABC transporters gene family.

Understanding tissue-specific expression patterns of PbrABC transporters will provide a more nuanced picture of their biological roles. In the case of pear, our analysis of previously obtained RNA-seq data has unveiled diverse expression patterns exhibited by PbrABC across distinct tissues (Fig. 5). Interestingly, different PbrABC family members exhibit distinct expression profiles. The diversity in these expression patterns of *PbrABC*s highlights the intricate regulatory mechanisms involved in various tissues of pear, emphasizing the complexity of ABC gene regulation. Additionally, the identification of specific expression patterns is noteworthy, such as the specificity observed in seven genes expressed uniquely in pear pollen and nine genes showing tissue-specific expression in leaves. The diversity observed in these unique expression patterns strongly indicates the multifaceted roles played by PbrABC transporters across different stages of pear growth and development.

Drought and salt stress are two major abiotic stressors that significantly impact plant growth and crop productivity. Several studies have provided compelling evidence of the involvement of specific ABC transporters in regulating plant responses to abiotic stress, particularly drought and salt stress. For instance, the Arabidopsis ABCG22, 25 and 40 transporters (also known as *AtABCG22*, *AtABCG25* and *AtABCG40*) has been shown to mediate abscisic acid (ABA) transport, a critical regulator of stomatal closure during drought stress [45–48]. Furthermore, the Arabidopsis ABCC transporters, AtABCC1 and AtABCC2, have garnered attention for their roles in ion detoxification and sequestration [29]. Additionally, knockout of *OsABCG36* resulted in increased Cd accumulation in root cell sap and enhanced Cd sensitivity in rice [20], indicating the biological function of OsABCG36 in cadmium tolerance. To better understand the potential functions of PbrABC transporters in pear responses to abiotic stresses, we examined the cis-element distribution in promoter regions. In the present study, we identified the *cis*-acting elements in promoter regions contained a variety of components involved in the stress response (drought response, low-temperature response, and defense and stress response) and phytohormone responses (gibberellin, auxin, abscisic acid, salicylic acid, and methyl jasmonate) (Fig. 4). These results indicated the potential functions the PbrABC transporters in environmental stress and plant development. Furthermore, we explored the expression patterns of PbrABC transporters after drought stress and salt stress treatment. A transcriptome analysis revealed that a large number of *PbrABC* genes were up-regulated in leaf tissues after drought or salt treatment (Fig. 6 and 7). For example, *PbrABCC9*, *PbrABCC14*, *PbrABCC21*, *PbrABCD7* and *PbrABCF1* are continuously up-regulated after salt treatment, 17 *PbrABC* genes decreased first under drought treatment, then increased after water recovery. Additionally, except for up-regulated genes, some PbrABC transporters were down-regulated in response to drought or salt stress. This dynamic expression pattern may indicate that these particular PbrABC transporters could potentially function as positive or negative regulators in the context of drought or salt stress, previous research confirmed this phenomenon in other species. For example, In Arabidopsis, AtABCB40, also known as AtPDR12, is an exemplary case of a positive regulator in drought stress responses [45]. Its overexpression enhances drought tolerance by mediating the efflux of stress-related compounds, thereby reducing ion accumulation and preventing stress-induced damage [45]. In addition, overexpression of AtABCG36 improves drought and salt stress resistance [49]. In rice, under salt stress, five genes (*OsABCF5*, *OsABCG27*, *OsABCG34*, *OsABCG36*, and *OsABCG45*) were up-regulated, and four genes (*OsABCB21*, *OsABCC17*, *OsABCG16*, and *OsABCG17*) were down-regulated harbors genes, suggesting that these ABC transporters may be involved in salt tolerance [50]. The intricate regulatory roles of specific ABC transporters in salt and drought stress responses highlight the complexity of plant adaptation to abiotic stressors. These transporters influence ion transport, osmotic adjustment, and the balance of stress-related hormones, collectively shaping the plant’s ability to cope with adverse environmental conditions.

However, it is crucial to recognize that the regulatory mechanisms of ABC transporters are not yet fully understood. Future research endeavors should aim to unravel the intricacies of these mechanisms, including their interactions with other stress-responsive genes and signaling pathways. Additionally, the potential for harnessing ABC transporters to improve stress tolerance in crops holds significant promise for sustainable agriculture.

## Conclusion

In summary, A total of 177 ABC transporter genes were identified in pear genome, which were divided into seven subfamilies, including 8 ABCAs, 40 ABCBs, 24 ABCCs, 8 ABCDs, 9 ABCEs, 8 ABCFs, and 80 ABCGs. Duplication analysis showed that WGD and dispersed duplication contributed to the expansion of the PbrABC gene family. Cis-regulatory element analysis of *PbrABC* promoters indicated associations with phytohormones and stress responses. Furthermore, tissue expression pattern and expression profile analyses under salt and drought stress indicated the biological functions of PbrABC transporter genes involved in the development and response to abiotic stresses. Overall, these findings provide a theoretical basis for further research biological roles of the PbrABC genes in pear.

## Materials and methods

### Plant materials and stress treatment

The “Duli” pear seeds used in this study were sourced from the pear germplasm orchard at the Pear Engineering Technology Research Center of Nanjing Agricultural University, located in Baima, Nanjing, with proper authorization. The seeds were initially placed on moistened gauze in a growth chamber under controlled conditions: temperature maintained at 25 ± 1 °C, darkness, and 60% relative humidity. After germination, the seedlings were transplanted into plastic pots and grown in a growth chamber for a period of five weeks, with a photoperiod of 16/8 hours and a temperature of 25 ± 1 °C. Subsequently, the seedlings were subjected to various stresses. For NaCl and drought stress, the seedlings were exposed to 200 mM NaCl and 20% PEG 6000, respectively. Pear leaves were systematically collected at specific time points following salt stress treatment, including 0 h, 4 h, 6 h, 12 h, 24 h, and 48 h. Additionally, for drought stress, the seedlings were sampled at continuous intervals of 0, 1, 3, 6 hours, and after 24 hours of rehydration. To preserve the samples, they were promptly frozen in liquid nitrogen and stored at − 80 °C.

### Genome-wide identification of ABC transporters in pear

To identify PbrABC transporters in pear, two methods were applied. First, 130 reference protein sequences of ABC transporter of *Arabidopsis thaliana* from the TAIR software (https://www.arabidopsis.org/) were used as query with the BLAST tool against the pear genome (http://pearomics.njau.edu.cn/). Then, the Hidden Markov Model of the PF00005 domain from the Pfam database was used to obtain the candidate PbrABC genes in pear [51]. After removing redundancy sequence, the putative PbrABC candidates were further verified using SMART (http://smart.embl-heidelberg.de/) [52], and Pfam search (http://pfam.xfam.org/search) [53]. As the culmination of our systematic exploration, we successfully pinpointed a total of 177 PbrABC transporter genes in pear genome.

### Phylogenetic analyses and conserved motif determination

The ABC protein sequences respectively obtained from Arabidopsis and pear genome. To construct phylogenetic trees for Arabidopsis and pear ABC transporters, several amino acid sequences were aligned using the ClustalX 2.0 program. Following this alignment, we constructed phylogenetic trees using Neighbor-Joining (NJ) method with the Poisson model and 1000 bootstrap replications [54]. The MEME online tool (http://meme-suite.org/) was used to determine the conserved motifs of PbrABC transporters [55]. TB tools software was then used for the motif visualization [56, 57].

### Chromosomal distribution and gene duplication of the PbrABC genes

To determine location information of ABC transporter genes, chromosome positions of all ABC transporter genes were confirmed in the pear database. Additionally, the gene duplication analysis was identified using the MCScanX software [58]. The circos project (http://circos.ca/) mapped the chromosomal location and the synteny relationships using TB tools software.

### Analysis of *cis*-acting elements in the promoter region of pear ABC family genes

To examine the role of the PbrABC gene’s regulatory region in pear, an upstream sequence within a 2000 bp distance from the start codon was retrieved from pear genome [44]. The promoter region sequences were analyzed for the presence cis-acting regulatory elements (CAREs) using the PlantCARE program (http://bioinformatics.psb.ugent.be/webtools/plantcare/html/) [59], and then TBtools was used for visual analysis.

### Expression profiling of ABC transporter genes in different tissues of pear

The expression levels of PbrABC genes in different tissues were analyzed through RNA-seq data, obtained from our previously published studies and unpublished data [44, 60, 61], including pollen, seed, petal, sepal, ovary, stem, bud, leaf and fruit (80 days after full blooming). The raw RNA-seq reads were cleaned by removing low-quality reads (quality score < 15), poly (A/T) tails, and adapter sequences. Finally, the values of fragments per kilobase million (FPKM) were used to indicate the expression levels of *PbrABC* genes. The heatmap of PbrABC gene expression was visualized using TB tools software. The RPKM values of PbrABC genes in different tissues of pear were shown in Table S2.

### Expression profiling of PbrABC transporter genes under drought or salt stress

RNA-seq data for PbrABC transporter gene under drought or salt stress was obtained from our lab. The sample for salt stress were taken at 0, 4, 6, 12, 24 and 48 h after sodium chloride (NaCl) treatment. The sample for drought stress were taken at 0, 1, 3, 6 and rehydration for 24 hours after drought treatment. Log transformation was carried out for reading/kilobase/million mapped values, and heatmap was constructed using software package TB tools. The RPKM values of PbrABC genes under salt and drought stress were respectively shown in Table S3 and S4.

### Quantitative real-time PCR (qRT-PCR)

Following the outlined protocol for total RNA extraction and genomic DNA contamination elimination, we designed qRT-PCR primers using Primer 5.0. The qRT-PCR was performed on the LightCycler-480 Detection System (Roche, Penzberg, Germany) using AceQ® qPCR SYBR® Green Master Mix (Vazyme, Nanjing, China). The qRT-PCR protocol included an initial 5-minute phase at 95 °C, followed by 45 cycles of 3 seconds at 95 °C, 10 seconds at 60 °C, and 30 seconds at 72 °C.

To ensure experimental rigor, all qRT-PCR analyses were carried out with strict adherence to three biological and technical replicates. PbrTUB were selected as reference genes for qRT-PCR using the 2^-ΔΔCt^ method [62]. It’s noteworthy that all steps, from RNA extraction to cDNA synthesis and subsequent analyses, were performed in triplicate. Refer to Table S5 for a comprehensive list of primers used in this assay.

### Supplementary Information

**Additional file 1: Table S1** Detailed information of the ABC transporter gene family in pear

**Additional file 2: Table S2** The RPKM values of PbrABCs in different tissues of pear

**Additional file 3: Table S3** The RPKM values of PbrABCs under salt stress

**Additional file 4: Table S4** The RPKM values of PbrABCs under drought stress

**Additional file 5: Table S5** The primers of PbrABC transporter genes for qRT-PCR

**Additional file 6: Fig. S1** The duplication modes of PbrABC transporter genes in pear. WGD: whole-genome duplication; TD: tandem duplication; PD: proximal duplication; DSD: dispersed duplication

## Data Availability

In the present study, the genome sequences and annotation files for the Chinese white pear from the Nanjing Agricultural University pear genome project website (http://peargenome.njau.edu.cn). All data generated or analyzed in this study are provided in the published article and its supplementary information files.

## References

[1] Higgins CF (2001). ABC transporters. physiology, structure and mechanism--an overview. Res Microbiol.

[2] Locher KP (2016). Mechanistic diversity in ATP-binding cassette (ABC) transporters. Nat Struct Mol Biol.

[3] Rees DC, Johnson E, Lewinson O (2009). ABC transporters: the power to change. Nat Rev Mol Cell Bio.

[4] Srikant S, Gaudet R (2019). Mechanics and pharmacology of substrate selection and transport by eukaryotic ABC exporters. Nat Struct Mol Biol.

[5] Xie T, Zhang Z, Fang Q, Du B, Gong X (2021). Structural basis of substrate recognition and translocation by human ABCA4. Nat Commun.

[6] Akhtar AA, Turner DPJ (2022). The role of bacterial ATP-binding cassette (ABC) transporters in pathogenesis and virulence: therapeutic and vaccine potential. Microb Pathogenesis.

[7] Higgins CF, Linton KJ (2004). The ATP switch model for ABC transporters. Nat Struct Mol Biol.

[8] Juan-Carlos P-DM, Perla-Lidia P-P, Stephanie-Talia M-M, Mónica-Griselda A-M, Luz-María T-E (2021). ABC transporter superfamily. An updated overview, relevance in cancer multidrug resistance and perspectives with personalized medicine. Mol Biol Rep.

[9] Lane TS, Rempe CS, Davitt J, Staton ME, Peng Y, Soltis DE, Melkonian M, Deyholos M, Leebens-Mack JH, Chase M (2016). Diversity of ABC transporter genes across the plant kingdom and their potential utility in biotechnology. BMC Biotechnol.

[10] Moore JM, Bell EL, Hughes RO, Garfield AS (2023). ABC transporters: human disease and pharmacotherapeutic potential. Trends Mol Med.

[11] Salam LB, Obayori OS (2023). Functional characterization of the ABC transporters and transposable elements of an uncultured Paracoccus sp. recovered from a hydrocarbon-polluted soil metagenome. Folia Microbiol.

[12] Dhara A, Raichaudhuri A (2021). ABCG transporter proteins with beneficial activity on plants. Phytochemistry.

[13] Do THT, Martinoia E, Lee Y (2018). Functions of ABC transporters in plant growth and development. Curr Opin Plant Biol.

[14] Li P, Luo T, Pu X, Zhou Y, Yu J, Liu L (2021). Plant transporters: roles in stress responses and effects on growth and development. Plant Growth Regul.

[15] Genovese I, Ilari A, Assaraf YG, Fazi F, Colotti G (2017). Not only P-glycoprotein: amplification of the ABCB1-containing chromosome region 7q21 confers multidrug resistance upon cancer cells by coordinated overexpression of an assortment of resistance-related proteins. Drug Resist Update.

[16] Robey RW, Shukla S, Finley EM, Oldham RK, Barnett D, Ambudkar SV, Fojo T, Bates SE (2008). Inhibition of P-glycoprotein (ABCB1) and multidrug resistance-associated protein 1 (ABCC1) mediated transport by the orally administered inhibitor, CBT-1((R)). Biochem Pharmacol.

[17] Vaidyanathan A, Sawers L, Gannon A-L, Chakravarty P, Scott AL, Bray SE, Ferguson MJ, Smith G (2016). ABCB1 (MDR1) induction defines a common resistance mechanism in paclitaxel- and olaparib-resistant ovarian cancer cells. Brit J Cancer.

[18] Golin J, Ambudkar SV, Gottesman MM, Habib AD, Sczepanski J, Ziccardi W, May L (2003). Studies with novel Pdr5p substrates demonstrate a strong size dependence for xenobiotic efflux*. J Biol Chem.

[19] Harris A, Wagner M, Du D, Raschka S, Nentwig L-M, Gohlke H, Smits SHJ, Luisi BF, Schmitt L (2021). Structure and efflux mechanism of the yeast pleiotropic drug resistance transporter Pdr5. Nat Commun.

[20] Fu S, Lu Y, Zhang X, Yang G, Chao D, Wang Z, Shi M, Chen J, Chao D-Y, Li R (2019). The ABC transporter ABCG36 is required for cadmium tolerance in rice. J Exp Bot.

[21] Christie JM, Yang H, Richter GL, Sullivan S, Thomson CE, Lin J, Titapiwatanakun B, Ennis M, Kaiserli E, Lee OR (2011). phot1 inhibition of ABCB19 primes lateral auxin fluxes in the shoot apex required for phototropism. PLoS Biol.

[22] Jenness MK, Tayengwa R, Murphy AS (2020). An ATP-binding cassette transporter, ABCB19, regulates leaf position and morphology during phototropin1-mediated blue light responses. Plant Physiol.

[23] Okamoto K, Ueda H, Shimada T, Tamura K, Koumoto Y, Tasaka M, Morita MT, Hara-Nishimura I (2016). An ABC transporter B family protein, ABCB19, is required for cytoplasmic streaming and gravitropism of the inflorescence stems. Plant Signal Behav.

[24] Titapiwatanakun B, Blakeslee JJ, Bandyopadhyay A, Yang H, Mravec J, Sauer M, Cheng Y, Adamec J, Nagashima A, Geisler M (2009). ABCB19/PGP19 stabilises PIN1 in membrane microdomains in Arabidopsis. Plant J.

[25] Kim K, Choi BY, Kang J, Shim D, Martinoia E, Lee Y (2022). Arabidopsis ABCG27 plays an essential role in flower and leaf development by modulating abscisic acid content. Physiol Plantarum..

[26] Bhati KK, Alok A, Kumar A, Kaur J, Tiwari S, Pandey AK (2016). Silencing of ABCC13 transporter in wheat reveals its involvement in grain development, phytic acid accumulation and lateral root formation. J Exp Bot.

[27] Chang Z, Jin M, Yan W, Chen H, Qiu S, Fu S, Xia J, Liu Y, Chen Z, Wu J (2018). The ATP-binding cassette (ABC) transporter OsABCG3 is essential for pollen development in rice. Rice.

[28] Feng T, He X, Zhuo R, Qiao G, Han X, Qiu W, Chi L, Zhang D, Liu M (2020). Identification and functional characterization of ABCC transporters for cd tolerance and accumulation in Sedum alfredii Hance. Sci Rep.

[29] Park J, Song WY, Ko D, Eom Y, Hansen TH, Schiller M, Lee TG, Martinoia E, Lee Y (2012). The phytochelatin transporters AtABCC1 and AtABCC2 mediate tolerance to cadmium and mercury. Plant J.

[30] Fu X, Liu H, Hassani D, Peng B, Yan X, Wang Y, Wang C, Li L, Liu P, Pan Q (2020). AaABCG40 enhances artemisinin content and modulates drought tolerance in *Artemisia annua*. Front Plant Sci.

[31] Chen N, Song B, Tang S, He J, Zhou Y, Feng J, Shi S, Xu X (2018). Overexpression of the ABC transporter gene TsABCG11 increases cuticle lipids and abiotic stress tolerance in Arabidopsis. Plant Biotechnol Rep.

[32] Qiao X, Li Q, Yin H, Qi K, Li L, Wang R, Zhang S, Paterson AH (2019). Gene duplication and evolution in recurring polyploidization–diploidization cycles in plants. Genome Biol.

[33] Biłas R, Szafran K, Hnatuszko-Konka K, Kononowicz AK (2016). Cis-regulatory elements used to control gene expression in plants. Plant Cell.

[34] Wittkopp PJ, Kalay G (2012). Cis-regulatory elements: molecular mechanisms and evolutionary processes underlying divergence. Nat Rev Genet.

[35] Gräfe K, Schmitt L (2021). The ABC transporter G subfamily in *Arabidopsis thaliana*. J Exp Bot.

[36] Mentewab A, Stewart CN (2005). Overexpression of an Arabidopsis thaliana ABC transporter confers kanamycin resistance to transgenic plants. Nat Biotechnol.

[37] Shi M, Wang S, Zhang Y, Wang S, Zhao J, Feng H, Sun P, Fang C, Xie X (2020). Genome-wide characterization and expression analysis of ATP-binding cassette (ABC) transporters in strawberry reveal the role of FvABCC11 in cadmium tolerance. Sci Hortic.

[38] Khan N, You FM, Datla R, Ravichandran S, Jia B, Cloutier S (2020). Genome-wide identification of ATP binding cassette (ABC) transporter and heavy metal associated (HMA) gene families in flax (*Linum usitatissimum* L.). BMC Genomics.

[39] Guo Z, Yuan X, Li L, Zeng M, Yang J, Tang H, Duan C (2022). Genome-wide analysis of the ATP-binding cassette (ABC) transporter family in *Zea mays* L. and its response to heavy metal stresses. Int J Mol Sci.

[40] Qiao Y, Jie Chen Z, Liu J, Nan Z, Yang H (2022). Genome-wide identification of *Oryza sativa*: a new insight for advanced analysis of ABC transporter genes associated with the degradation of four pesticides. Gene..

[41] Sánchez-Fernández R, Davies TG, Coleman JO, Rea PA (2001). The *Arabidopsis thaliana* ABC protein superfamily, a complete inventory. J Biol Chem.

[42] Garcia O, Bouige P, Forestier C, Dassa E (2004). Inventory and comparative analysis of rice and arabidopsis ATP-binding cassette (ABC) systems. J Mol Biol.

[43] Ofori PA, Mizuno A, Suzuki M, Martinoia E, Reuscher S, Aoki K, Shibata D, Otagaki S, Matsumoto S, Shiratake K (2018). Genome-wide analysis of ATP binding cassette (ABC) transporters in tomato. PLoS One.

[44] Wu J, Wang Z, Shi Z, Zhang S, Ming R, Zhu S, Khan MA, Tao S, Korban SS, Wang H (2013). The genome of the pear (*Pyrus bretchneideri* Rehd.). Genome Res.

[45] Kang J, Hwang JU, Lee M, Kim YY, Assmann SM, Martinoia E, Lee Y (2010). PDR-type ABC transporter mediates cellular uptake of the phytohormone abscisic acid. P Natl Acad Sci USA.

[46] Kuromori T, Miyaji T, Yabuuchi H, Shimizu H, Sugimoto E, Kamiya A, Moriyama Y, Shinozaki K (2010). ABC transporter AtABCG25 is involved in abscisic acid transport and responses. P Natl Acad Sci USA.

[47] Kuromori T, Sugimoto E, Shinozaki K (2011). Arabidopsis mutants of AtABCG22, an ABC transporter gene, increase water transpiration and drought susceptibility. Plant J.

[48] Ying W, Liao L, Wei H, Gao Y, Liu X, Sun L (2023). Structural basis for abscisic acid efflux mediated by ABCG25 in Arabidopsis thaliana. Nat Plants.

[49] Kim DY, Jin JY, Alejandro S, Martinoia E, Lee Y (2010). Overexpression of AtABCG36 improves drought and salt stress resistance in Arabidopsis. Physiol Plantarum.

[50] Nguyen VNT, Moon S, Jung K-H (2014). Genome-wide expression analysis of rice ABC transporter family across spatio-temporal samples and in response to abiotic stresses. J Plant Physiol.

[51] Yoon BJ (2009). Hidden Markov models and their applications in biological sequence analysis. Curr Genomics.

[52] Ponting CP, Schultz J, Milpetz F, Bork P (1999). SMART: identification and annotation of domains from signalling and extracellular protein sequences. Nucleic Acids Res.

[53] Mistry J, Chuguransky S, Williams L, Qureshi M, Salazar GA, Sonnhammer ELL, Tosatto SCE, Paladin L, Raj S, Richardson LJ (2021). Pfam: the protein families database in 2021. Nucleic Acids Res.

[54] Kumar S, Stecher G, Tamura K (2016). MEGA7: molecular evolutionary genetics analysis version 7.0 for bigger datasets. Mol Biol Evol.

[55] Bailey TL, Boden M, Buske FA, Frith M, Grant CE, Clementi L, Ren J, Li WW, Noble WS (2009). MEME SUITE: tools for motif discovery and searching. Nucleic Acids Res.

[56] Chen C, Chen H, Zhang Y, Thomas HR, Frank MH, He Y, Xia R (2020). TBtools: an integrative toolkit developed for interactive analyses of big biological data. Mol Plant.

[57] Chen C, Wu Y, Li J, Wang X, Zeng Z, Xu J, Liu Y, Feng J, Chen H, He Y (2023). TBtools-II: a “one for all, all for one” bioinformatics platform for biological big-data mining. Mol Plant.

[58] Wang Y, Tang H, Debarry JD, Tan X, Li J, Wang X, Lee TH, Jin H, Marler B, Guo H (2012). MCScanX: a toolkit for detection and evolutionary analysis of gene synteny and collinearity. Nucleic Acids Res.

[59] Rombauts S, Déhais P, Van Montagu M, Rouzé P (1999). PlantCARE, a plant cis-acting regulatory element database. Nucleic Acids Res.

[60] Li Q, Qiao X, Yin H, Zhou Y, Dong H, Qi K, Li L, Zhang S (2019). Unbiased subgenome evolution following a recent whole-genome duplication in pear (*Pyrus bretchneideri* Rehd.). Hortic Res.

[61] Zhou H, Yin H, Chen J, Liu X, Gao Y, Wu J, Zhang S (2016). Gene-expression profile of developing pollen tube of *Pyrus bretchneideri*. Gene Expr Patterns.

[62] Schmittgen TD, Livak KJ (2008). Analyzing real-time PCR data by the comparative CT method. Nat Protoc.

