# Supplementary figures and images for: Identification and expression analysis of ATP-binding cassette (ABC) transporters revealed its role in regulating stress response in pear (Pyrus bretchneideri)
Source: BMC Genomics. 2024 Feb 12;25:169. doi: 10.1186/s12864-024-10063-1 (PMC10863237; doi:10.1186/s12864-024-10063-1)

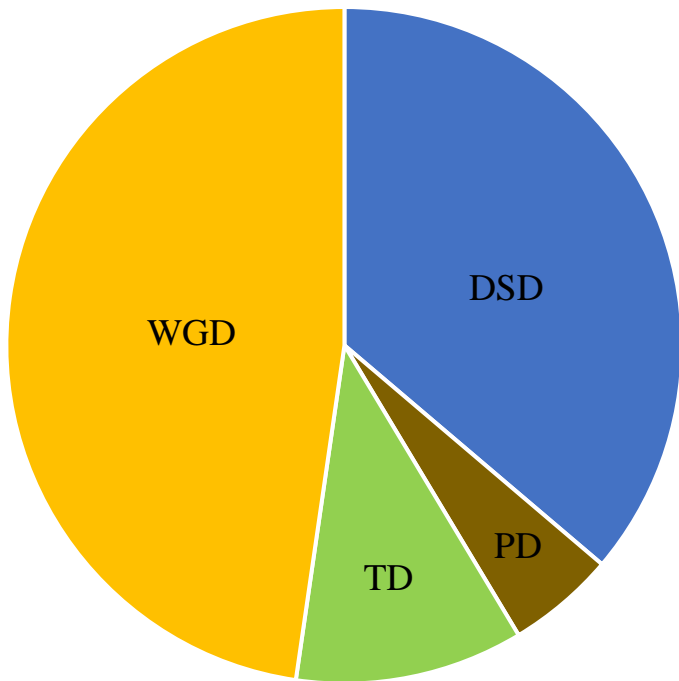

Supplement: Supplementary file 6 — Additional file 6: Fig. S1 The duplication modes of PbrABC transporter genes in pear. WGD: whole-genome duplication; TD: tandem duplication; PD: proximal duplication; DSD: dispersed duplication [file 12864_2024_10063_MOESM6_ESM.pdf]
